# Prospective, randomized, controlled, double-blind, multi-center, multinational study on the safety and efficacy of 6% Hydroxyethyl starch (HES) sOlution versus an Electrolyte solutioN In patients undergoing eleCtive abdominal Surgery: study protocol for the PHOENICS study

**DOI:** 10.1186/s13063-022-06058-6

**Published:** 2022-02-22

**Authors:** Wolfgang Buhre, Dianne de Korte-de Boer, Marcelo Gama de Abreu, Thomas Scheeren, Matthias Gruenewald, Andreas Hoeft, Donat R. Spahn, Alexander Zarbock, Sylvia Daamen, Martin Westphal, Ute Brauer, Tamara Dehnhardt, Sonja Schmier, Jean-Francois Baron, Stefan De Hert, Željka Gavranović, Bernard Cholley, Tomas Vymazal, Wojciech Szczeklik, Helmar Bornemann-Cimenti, Marina Blanca Soro Domingo, Ioana Grintescu, Radmilo Jankovic, Javier Belda

**Affiliations:** 1 Division of Acute and Critical Medicine, Maastricht University Medical Centre, Maastricht, The Netherlands; 2 Department of Anesthesiology and Pain Medicine, Maastricht University Medical Centre, Maastricht, The Netherlands; 3 Department of Anesthesiology and Intensive Care Medicine, Pulmonary Engineering Group, University Hospital Carl Gustav Carus, Technische Universität Dresden, Dresden, Germany; 4 Department of Intensive Care and Resuscitation, Anesthesiology Institute, Cleveland Clinic, Cleveland, OH USA; 5 Department of Outcomes Research, Anesthesiology Institute, Cleveland Clinic, Cleveland, OH USA; 6 Department of Anesthesiology, University Medical Center Groningen, Groningen, The Netherlands; 7 Department of Anesthesiology and Intensive Care Medicine, University Hospital Schleswig-Holstein, Kiel, Germany; 8 Department of Anesthesiology and Operative Intensive Care Medicine, University Hospital Bonn, Bonn, Germany; 9 Institute of Anesthesiology, University and University Hospital of Zürich, Zürich, Switzerland; 10 Anesthesiology, Intensive Care Medicine and OR Facilities, University and University Hospital of Zürich, Zürich, Switzerland; 11 Department of Anesthesiology, Intensive Care and Pain Medicine, University Hospital Muenster, Muenster, Germany; 12 European Society of Anaesthesiology and Intensive Care, Brussels, Belgium; 13 Fresenius Kabi Deutschland GmbH, Bad Homburg, Germany; 14 Department of Medical Scientific Affairs, Hospital Care Division, B. Braun Melsungen AG, Melsungen, Germany; 15 Department of Anesthesioloy and Perioperative Medicine, Gent University Hospital – Gent University, Ghent, Belgium; 16 Department of Anesthesiology and Intensive Care, University Hospital Center Sestre Milosrdnice, Zagreb, Croatia; 17 Service d’Anesthésie-Réanimation, Hôpital Européen Georges Pompidou, Paris, France; 18 Department of Anesthesiology and Intensive Medicine, University Hospital Motol, Prague, Czech Republic; 19 Department of Anaesthesiology and Intensive Therapy, 5th Military Clinical Hosptial, Krakow, Poland; 20 Department of Anesthesiology and Intensive Care Medicine, Medical University of Graz, Graz, Austria; 21 Department of Surgery, Clinic University Hospital, Valencia, Spain; 22 Department of Anesthesia, Reanimation and Pain Therapy, Clinic University Hospital, Valencia, Spain; 23 Clinic of Anaesthesia and Intensive Care Medicine, Clinical Emergency Hospital of Bucharest, Bucharest, Romania; 24 Department of Anaesthesia and Intensive Care Medicine, Faculty of Medicine, ‘Carol Davila’ University of Medicine and Pharmacy, Bucharest, Romania; 25 Clinic for Anesthesiology and Intensive Therapy, University Clinical Center Nis, School of Medicine, University of Nis, Nis, Serbia

**Keywords:** Volume therapy, Colloids, Hydroxyethyl starch, HES, Surgery, Blood loss, Multi-center, Multinational, Double-blinded, Randomized controlled trial, Non-inferiority trial, Safety

## Abstract

**Background:**

Hydroxyethyl starch (HES) solutions are used for volume therapy to treat hypovolemia due to acute blood loss and to maintain hemodynamic stability. This study was requested by the European Medicines Agency (EMA) to provide more evidence on the long-term safety and efficacy of HES solutions in the perioperative setting.

**Methods:**

PHOENICS is a randomized, controlled, double-blind, multi-center, multinational phase IV (IIIb) study with two parallel groups to investigate non-inferiority regarding the safety of a 6% HES 130 solution (Volulyte 6%, Fresenius Kabi, Germany) compared with a crystalloid solution (Ionolyte, Fresenius Kabi, Germany) for infusion in patients with acute blood loss during elective abdominal surgery. A total of 2280 eligible patients (male and female patients willing to participate, with expected blood loss ≥ 500 ml, aged > 40 and ≤ 85 years, and ASA Physical status II–III) are randomly assigned to receive either HES or crystalloid solution for the treatment of hypovolemia due to surgery-induced acute blood loss in hospitals in up to 11 European countries. The dosing of investigational products (IP) is individualized to patients’ volume needs and guided by a volume algorithm. Patients are treated with IP for maximally 24 h or until the maximum daily dose of 30 ml/kg body weight is reached.

The primary endpoint is the treatment group mean difference in the change from the pre-operative baseline value in cystatin-C-based estimated glomerular filtration rate (eGFR), to the eGFR value calculated from the highest cystatin-C level measured during post-operative days 1-3. Further safety and efficacy parameters include, e.g., combined mortality/major post-operative complications until day 90, renal function, coagulation, inflammation, hemodynamic variables, hospital length of stay, major post-operative complications, and 28-day, 90-day, and 1-year mortality.

**Discussion:**

The study will provide important information on the long-term safety and efficacy of HES 130/0.4 when administered according to the approved European product information. The results will be relevant for volume therapy of surgical patients.

**Trial registration:**

EudraCT 2016-002162-30. ClinicalTrials.govNCT03278548

## Background

Hypovolemia is characterized by reduced circulating blood volume, which can be caused by a number of events, including blood loss due to surgery or trauma. Substantial loss of intravascular volume may lead to hemodynamic instability, tissue hypoperfusion, cellular hypoxia, organ damage, and ultimately death [1]. Accordingly, treatment aims at controlling further blood loss and restoring physiologic organ perfusion by providing infusion solutions targeting to match oxygen demands with delivery. While fluid therapy aims to substitute for protein-free fluid losses, the goal of volume therapy is substitution of blood volume, i.e., treatment of hypovolemia to maintain hemodynamics and vital functions [2, 3]. Crystalloid solutions (composed of water and electrolytes) and colloid solutions (containing macromolecules such as hydroxyethyl starch (HES), gelatin, or albumin) are routinely used for volume therapy [4]. While crystalloid solutions diffuse easily into the interstitial space, colloid solutions contain macromolecules, which are unable to pass intact semi-permeable biological membranes. In the surgical setting, an increased volume effect of colloids is observed compared to crystalloids [5–10]. This volume sparing effect is expected to reduce edema and associated complications [6–9].

The most relevant indication for the administration of colloids is volume replacement in patients undergoing general surgical procedures [6, 7]. Volume replacement during surgery aims for prompt and goal-directed colloid administration to optimize hemodynamic variables and to prevent fluid overload. Perioperative volume therapy should be guided by treatment algorithms that primarily use flow- or pressure-based target variables for hemodynamic optimization [9]. It has been demonstrated that post-operative outcomes may be improved with an algorithm-guided fluid administration; however, data are sparse [10].

HES represents one of the most frequently used colloids for volume replacement [11]. The HES-containing solution administered in this study, Volulyte 6% (HES 130/0.4), features a mean molecular weight of 130 kDa, a molar substitution of 0.38–0.45, and a substitution pattern of approximately 8:12.

Following the publication of investigator-initiated trials with methodological limitations indicating renal impairment and increased mortality upon HES administration in critically ill patients [12–14], the European Medicines Agency (EMA) started procedures to analyze the benefits and risks of HES-containing solutions. An Article 31 referral procedure (EMEA/H/A-31/1348) and an urgent union procedure under Article 107i of Directive 2001/83/EC (EMEA/H/A-107i/1376) for HES-containing medicinal products were initiated in 2012 and completed in 2013.

As part of the outcome of these referral procedures, the Marketing Authorization Holders of HES-containing solutions were requested to conduct, amongst others, a phase IV clinical trial to demonstrate the long-term safety of HES-containing solutions with regard to renal failure and mortality as well as efficacy in the perioperative setting. Scientific advice was given by the EMA’s Scientific Advice Working Party (SAWP) and adopted by the Committee for Medicinal Products for Human Use (CHMP) (EMA/CHMP/SAWP/544745/2014) regarding the design of this study. The current trial aims to study the elective abdominal surgery patients (> 40–≤ 85 years of age, ASA II–III) with an increased risk for post-operative complications. Administration of HES will be performed in accordance with the recently approved European HES product information, which is partly different to the volume regimen in the recently published FLASH study [15]. In the latter study, HES was used in patients with known risk factors (defined by an acute kidney risk index as an inclusion criterion) for the development of renal failure, and the dose limitations were not adhered to in a significant subset of patients. Consequently, 24% of patients in the HES group and 23% in the saline group had mild or moderate kidney dysfunction despite being a contraindication. In a recent multi-centric study in patients undergoing elective surgery, no signs for increased incidence of renal failure were found [16]. Moreover, a lower post-operative morbidity survey score was reported in a small-scale study when applying a goal-directed fluid management approach in surgical patients [17].

### Study objective

The primary objective of this study is to assess non-inferiority regarding the safety of a 6% HES solution compared to an electrolyte solution in patients with acute blood loss during elective abdominal surgery. Secondary objectives are to further assess the efficacy and safety, e.g., combined mortality/major complications until day 90, renal function, coagulation parameters, inflammation, hemodynamic variables, length of hospital stay, major post-operative complications, and 28-day, 90-day, and 1-year mortality.

## Methods/design

### Trial design

PHOENICS is a prospective, randomized, controlled, double-blind, multi-center, multinational phase IV study (in the Czech Republic and Serbia phase IIIb) performed in two parallel groups aiming to assess the safety of a 6% HES solution (Volulyte 6%, Fresenius Kabi Deutschland GmbH, Germany) versus an electrolyte solution (Ionolyte, Fresenius Kabi Deutschland GmbH, Germany) for the treatment of hypovolemia caused by acute blood loss in elective abdominal surgery. A total of 2280 eligible patients are randomly assigned to receive either Volulyte 6% (HES group) or Ionolyte (crystalloid group) in a 1:1 ratio, stratified by site. To achieve normovolemia, volume therapy will be given according to the clinical algorithms defined by each site before patient recruitment.

### Participants

The study is conducted in a population of adult male and female patients > 40 and ≤ 85 years of age undergoing elective abdominal surgery with an expected blood loss of ≥ 500 ml. Eligible patients are patients with an ASA Physical status II–III and have to provide signed written informed consent to participate in this study. Women of childbearing potential must be tested negative for pregnancy (urine or serum) before inclusion. Reasons for exclusion are hypersensitivity to the active substances or to any of the other excipients of the investigational medicinal products, body weight ≥ 140 kg, sepsis, burns, renal impairment (AKIN stage ≥ 1 or chronic), acute and/or chronic renal replacement therapy (RRT), intracranial or cerebral hemorrhage, critically ill patients (typically admitted to the intensive care unit), hyperhydration, pulmonary edema, dehydration, hyperkalemia, severe hypernatremia, severe hyperchloremia, severely impaired hepatic function, congestive heart failure, severe coagulopathy, organ transplant patients, metabolic alkalosis, and simultaneous participation in another interventional clinical trial (drugs or medical devices studies). It is planned to activate study sites in up to 11 countries. A list of all participating sites is accessible at ClinicalTrials.gov.

### Investigational products (IPs)

The investigational test product Volulyte 6% (Fresenius Kabi Deutschland GmbH, Germany) is a clear to slightly opalescent, colorless to slightly yellow (trial samples are colorless) 6% 130/0.4 hydroxyethyl starch solution in an isotonic, fully balanced electrolyte solution. The investigational reference product Ionolyte (Fresenius Kabi Deutschland GmbH, Germany) is a clear and colorless, aqueous, fully balanced electrolyte solution. Ionolyte is considered a suitable comparator since it has the identical electrolyte composition as Volulyte 6%. Both products are licensed solutions for infusion provided in a 500-ml polyolefin bag (freeflex) with overwrap.

### Study phases

#### Enrollment (screening, randomization, and baseline)

Patients are screened within 1 week before surgery including verification of in- and exclusion criteria, demographic data, medical history, and anamnesis as well as the main indication for surgery, provision of informed consent, and pregnancy test in women of childbearing potential. The study starts with randomization (maximum of 1 day prior to surgery). After randomization and prior to induction of anesthesia, baseline variables will be determined (see Table 1).

**Table 1:** PHOENICS study flow diagram

Procedure	Time										
	Screening: within 1 week before surgery	Randomization: max. 1 day prior to surgery	T0 (baseline): after randomization and prior induction of anesthesia	T1: during surgery	T2: end of surgery	T3: POD 1 (morning)	T4: POD 2 and 3 (morning)	T5: POD 4 until POD 7 or hospital discharge, whatever occurs first (morning)	T6: POD 8 until POD 10 or hospital discharge, whatever occurs first (morning)	T7^**1)**^/T8^**2)**^ : 28/90 days after surgery	T9^**3)**^ 1 year after surgery
Informed consent	X										
Inclusion/exclusion criteria	X										
Randomization		X									
Demographic data	X										
Concomitant diseases (only ongoing and relevant resolved) and anamnestic baseline characteristics reflecting the surgical risk	X										
Date of hospital admission	X										
Main diagnosis for surgery	X										
Type of anesthesia				X							
Type of surgery					X						
Time of skin incision				X							
Time of skin suture					X						
Fluid input (colloids, crystalloids) in the 24 h prior IP treatment start			X								
Temperature [°C]			X		X	X	X				
Cystatin-C [mg/l] Cystatin-C-based eGFR [ml/min] Cystatin-C-based mean eGFR (calculated from the highest cystatin-C level during days 1–10) [ml/min] SCr [mg/dl] SCr-based eGFR [ml/min] AKIN score (calculated) RIFLE score (calculated) Urine output (if available)			X		X	X	X	Only cystatin C-based mean GFR	Only cystatin C-based mean GFR	X (without urine output)	
C-reactive protein [mg/l]			X		X	X					
Platelet count [μ/l] INR aPTT [s]			X		X	X					
Na^+^ [mmol/l] K^+^ [mmol/l] Ca^2+^ [mmol/l] Cl^-^ [mmol/l]			X		X	X					
pCO_2_ [mmHg] pO_2_ [mmHg] HCO_3_^−^ [mmol/l] SaO_2_ [%] pH Base excess [mEq/l]			X		X						
Hb [g/dl] Hct [%]			X		X	X	X				
Lactate [mmol/l]			X		X	X	X				
ScvO_2_ [%] (if available)			X		X	X					
Administered IP volume [ml]				During the duration of IP administration (max 24 h)							
Fluid input [ml] (every i.v. medication, applied blood products) Fluid output [ml] (drainage, urine output, estimated intra-operative blood loss)			Cumulative from T0 until T4								
Estimated intra-operative blood loss [ml]					X						
MAP [mmHg] HR [beats/min]			X	X*	X	X^#^	X^#^				
SAP [mmHg] DAP [mmHg] CVP [mmHg] (if available)			X	X*	X						
Hemodynamics as required to determine volume responsiveness (at least one variable) • MAP [mmHg] • SV [ml] • SVV [%] • PPV [%] • SVI [ml/min^2^]				**During duration of IP administration to assess volume responsiveness							
Major post-operative complications (including renal)						X	X	X	X	X	
Date and time of hospital discharge (if applicable)						At hospital discharge				X	
Fulfilment of fit for discharge from hospital criteria (PADS)						Daily until fulfillment				X	
Transfer to ICU/IMC					X						
Date of ICU admission (if applicable)						At ICU admission					
Date and time of discharge from ICU (if applicable)						At ICU discharge				X	
Fit for ICU discharge criteria (if applicable)						Daily until fulfillment				X	
Use of renal replacement therapy (RRT)					X	X	X	X			
Mechanical ventilation			X	X	X	X	X	X			
Vasoactive/inotropic drugs • Administered drug • Dosage/volume			Continuously								
Applied blood products • Administered drug • Dosage/volume				Continuously							
Crystalloid/albumin • Administered drug • Dosage/volume				Whenever applied							
Antibiotics Contrast agents Diuretics Basal infusion (volume)			Whenever applied								
(Serious) adverse events			Continuously								
New RRT										X	X
Mortality (in-hospital/out of hospital) and cause					X	X	X	X	X	X	X
Study termination					At termination						

*At least every 30 min

**As required

^#^MAP if available

^1)^± 5 days

^2)^± 14 days

^3)^± 30 days

#### Treatment phase

During the treatment phase, IP is administered intravenously to treat hypovolemia caused by surgery-induced acute blood loss. The administration of the IP and all defined safety and efficacy parameters are documented in an electronic case record form (see Tables 1 and 2). Perioperative IP administration has to be guided either by mean arterial pressure (MAP), stroke volume (SV), stroke volume variation (SVV), stroke volume index (SVI), or pulse pressure variation (PPV). The choice of the hemodynamic stabilization algorithm and the definition of volume responsiveness relies with the local investigator at each site at the beginning of the study but has to be followed for both groups during the whole study period within the study site.

**Table 2:** Secondary variables

Safety parameters	Efficacy parameters	Other variables
**Renal function** • Cystatin-C • Serum creatinine • Cystatin-C-based estimated glomerular filtration rate (eGFR) • Lowest cystatin-C-based eGFR PODs 1–10^1^ • Serum creatinine-based eGFR • AKIN and RIFLE score^2^ • Urine output^3^ **Coagulation** • Platelet count • International normalized ratio • Activated partial thromboplastin time **Inflammation** • C-reactive protein **Adverse events** • (Serious) adverse events/reactions **Calculated red blood cell loss** **Estimated intra-operative blood loss** **Outcome** • Composite of mortality and major post-operative complications (including renal) until day 90 • Length of stay (LOS): LOS in the hospital LOS in the intensive care unit^3^ Fit for discharge from ICU/hospital^4^ • Hours on mechanical ventilation • In-hospital/out of hospital mortality (including cause) • (New) renal replacement therapy (RRT)	**Fluid administration** • Administration of IP volume **Fluid balance** • Fluid input and output **Hemodynamics/vital signs** • Heart rate • Temperature • Mean arterial pressure (MAP) • Systolic arterial blood pressure • Diastolic arterial blood pressure • Central venous pressure^3^ *at least one of the following parameters (volume algorithm):* • Stroke volume (SV) • Stroke volume variation (SVV) • Stroke volume index (SVI) • Pulse pressure variation (PPV) • Mean arterial blood pressure (MAP) **Laboratory data** • Arterial blood gas analysis Partial pressure of carbon dioxide Partial pressure of oxygen Bicarbonate Arterial oxygen saturation pH Base excess Lactate Hemoglobin Hematocrit • Central venous oxygen saturation^3^ • Serum electrolytes Sodium Potassium Calcium Chloride **Major post-operative complications**^5^	**Demographic data and medical history** • Age • Gender • Height • Weight • Ethnicity • Concomitant diseases and anamnestic baseline characteristics reflecting the surgical risk • Fluid input in the 24 h prior IP treatment start **Surgery related data** • Main diagnosis leading to surgery • Type of anesthesia • Type of surgery • Time of skin incision/suture **Concomitant medication** • Antibiotic therapy • Contrast agents • Diuretics • Vasoactive/inotropic drugs • Blood products • Basal infusion administration • Crystalloid solutions/albumin

^1^Calculated from the highest cystatin-C level during days 1–10, or hospital discharge, whatever occurs first

^2^According to Bagshaw et al. 2008 [33]. Reference for the calculation of AKIN and RIFLE scores is the pre-operative creatinine value at baseline; missing baseline creatinine levels will be estimated [18]

^3^If applicable/if available

^4^According to Marshall et al. 1997 [19]

^5^Including renal, defined according to the statement of the ESA-ESICM joint taskforce on perioperative outcome [36]. The classifications of complications into “major” will be done if graded as moderate or severe

The administration of IP starts during surgery and stops as soon as the patient is hemodynamically stabilized, but not exceeding the maximum daily IP dose of 30 ml kg^−1^ or the maximum treatment duration of 24 h.

As HES preparations for volume replacement rarely cause allergic reactions of varying severity, the first 10–20 ml of IP is infused slowly. In case of an allergic reaction, the infusion is stopped immediately, and appropriate treatment will be initiated.

If the patient is hemodynamically not stabilized after completion of the treatment phase, crystalloid solutions and/or albumin are to be administered for further volume therapy. The choice of the respective solution is at the discretion of the treating physician and is documented in the electronic case report form (eCRF) including applied volume. If needed, transfusion of blood products can be performed throughout the study period; blood products should be given in accordance with the current ESA guideline, recommending a target hemoglobin concentration of 7–9 g/dl during active bleeding.

#### Daily assessments

Patients are examined daily, including blood sampling, starting at POD 1 in the morning until POD 10 or hospital discharge, whatever occurs first. Safety and efficacy variables are recorded (see Tables 1 and 2).

#### Follow-up (FU)

Patients are invited to the hospital for additional visits on day 28 (± 5 days) and day 90 (± 14 days) after surgery. The patients will be invited for these follow-up visits to the hospital, by a follow-up letter, e-mail, or call. Alternatively, a qualified person (nurse or physician) may visit the patient at home. At each of these visits, renal function by means of serum cystatin-C, serum creatinine, cystatin-C-based and/or serum creatinine-based estimated glomerular filtration rate (eGFR), AKIN, and RIFLE scores are assessed. In addition, the occurrence of new RRT, (serious) adverse events, major post-operative complications, mortality, length of stay (hospital and ICU), and fit for discharge are recorded (see Tables 1 and 2). One year after surgery (± 30 days), mortality and renal outcome (i.e., new RRT after the 90-day visit) are re-assessed via a follow-up telephone call. Study phases and assessments are summarized in the PHOENICS study flow diagram (Table 1).

### Concomitant medication

The following are the allowed concomitant medication/therapy:

- Medication that is clinically required* (except other volume replacement (colloids or crystalloids) therapy during IP treatment period) by decision of the treating physician.
- Vasoactive/inotropic treatment, starting earliest after third IP volume challenge.
- Basal infusion of crystalloid solution (up to 4 ml/kg/h) as required. The choice of the crystalloid for the basal infusion and the required infusion rate is made by the treating physician.
- Crystalloid solutions or albumin, if clinically required, after achieving the maximum daily dose of 30 ml/kg IP or maximum IP treatment period of 24 h, whatever occurs first.
- If concomitant blood products are necessary, these should only be given according to the most current version of the ESA guideline on the management of severe perioperative bleeding, recommending a target hemoglobin concentration of 7–9 g/dl during active bleeding [20].

Administration of concomitant medication has to be provided via a separate infusion system independently from the IP infusion system. * e.g. basal vasoactive medication and initial crystalloid infusion to compensate for the effects of anesthetics for hemodynamic stabilization after induction of anesthesia (e.g., low doses of vasopressors).

The following are not allowed concomitant medication:

- Any colloid (i.e., gelatin solutions, albumin, dextran, other HES solutions) during the treatment phase
- Synthetic colloids (i.e., gelatin solutions, dextran, and other HES solutions) after the treatment phase until hospital discharge
- Intravenous crystalloid solutions during treatment phase besides basal infusion (up to 4 ml^−1^kg^−1^ h^−1^)

### Outcomes

#### Primary outcome

The primary outcome is the mean difference in cystatin-C-based eGFR calculated from the highest cystatin-C level measured during PODs 1–3. This difference is calculated for the change from the pre-operative baseline eGFR value. Measurements of cystatin-C in serum are performed in a central laboratory, blinded for patient allocation. Cystatin-C-based eGFR is calculated based on the equation developed by Inker et al. [21].

#### Secondary outcomes

Secondary variables (listed in Table 2) will provide further information on the safety and efficacy of the 6% HES solution. The date and time of assessment are documented for all variables. The assessments and corresponding time points are summarized in the PHOENICS study flow diagram (Table 1).

Laboratory analyses are performed in local laboratories of the sites, except for cystatin-C, which is determined in a central laboratory for all centers.

The investigator must record all (serious) adverse events ((S)AEs) occurring during the study on the appropriate eCRF page. All SAEs except those exempted from expedite reporting must be reported to the sponsor within 24 h (one working day) of the investigator becoming first knowledge. The sponsor will notify the competent authorities, IECs, and all concerned investigators about suspected unexpected serious adverse reactions in line with pertinent legal requirements.

### Sample size

The primary objective of this study is to demonstrate non-inferiority of treatment with Volulyte 6% versus Ionolyte with respect to the prevention of relevant renal changes, quantified by the treatment group mean difference in change from the pre-operative baseline eGFR value to the eGFR value calculated from highest cystatin-C levels measured during post-operative days 1–3.

A *t*-test (*α* = 0.025 one-tailed) with a non-inferiority margin of *δ* = 8.1 mL/min/1.73 m^2^, corresponding to 9% of the lower limit of the normal range of GFR of 90mL/min/1.73 m^2^ (National Kidney Foundation 2002), was used for the sample size calculation. The EMA recommended in their final scientific advice (dated 22 January 2015) to use a non-inferiority margin lower than 10%. The standard deviation of the primary efficacy endpoint was assumed to be the same in both treatment arms and was estimated to be 52.5 mL/min/1.73 m^2^ based on data reported by Felicio and co-workers [22] and an estimated correlation coefficient of 0.5 for the correlation between pre-operative and post-operative eGFR values. It was further assumed that the real treatment group difference is zero.

Sample size estimation (power 1–*β*=0.9) resulted in 909 patients per group, i.e., 2280 patients in total, including a drop-out rate of 20% (SAS PROC POWER). This sample size also covers the secondary composite variable “mortality/major post-operative complications (including renal) until day 90.”

The drop-out rate is monitored during the study in order to adjust the sample size in case the drop-out rate exceeds 20%.

### Randomization, blinding, and unblinding

Assignment to study treatment is randomized in a 1:1 ratio, stratified by site. An Interactive Response Technology System (IRTS) is used for randomization of patients (random permuted blocks of variable size) and IP supply.

The treatment group randomization list and the IP kits list are generated prior to the initiation of the study by the IRTS vendor and approved by an unblinded statistician (not involved in the study data analyses). Eligible patients are enrolled by the investigator and randomized by the IRTS.

Investigators and medical staff as well as study participants are blinded to the study treatment. Emergency unblinding will only be done via the IRTS by an investigator and/or dedicated authorized personnel (e.g., Pharmacovigilance Department of the Sponsor).

### Statistical methods

All programming of tables, figures, listings, and statistical analyses will be performed using SAS® version 9.4 or higher. The planned statistics will be done in accordance with guideline ICH E9.

#### Primary endpoint

The mean difference in cystatin-C-based eGFR, calculated from the highest cystatin-C level measured during PODs 1–3, will be estimated with a two-sided 95% confidence interval by analyzing the change from the pre-operative baseline eGFR value in an analysis of covariance model, which includes treatment and study site as factors and baseline eGFR as a covariate. Non-inferiority of Volulyte 6% compared to Ionolyte will be tested with a one-sided contrast (*α* = 0.025) for a non-inferiority margin of *δ* = 8.1 mL/min/1.73 m^2^ (see the “Sample size” section for justification). The primary endpoint will be analyzed in the full analysis set (FAS) and in the per-protocol analysis set (PPS). Since the use of the FAS may not be conservative in a non-inferiority trial (ICH-E9 guideline) [23], the non-inferiority test performed in both datasets is considered as co-primary.

By definition, the FAS will comprise all patients reaching the post-operative period and monitored at least once with respect to the changes in cystatin-C-based eGFR. The results of both analyses (i.e., PPS and FAS) will be compared and possible differences assessed.

Secondary outcomes will be compared by means of descriptive statistics and appropriate statistical tests (time to event analyses (Kaplan-Meier plots, Cox regression), analysis of covariance, logistic regression, Mann-Whitney *U* test, *χ*^2^ test).

Strong efforts will be made to collect all data points in the study (e.g., by training of investigators and other authorized staff, regular monitoring of data entries). Missing pre-operative baseline values for serum creatinine-based eGFR will be imputed by assuming a numerical value at the lower end of the normal range (i.e., 75 ml/min per 1.73 m^2^). The corresponding missing serum creatinine value will be estimated based on the simplified “modification of diet in renal disease” formula based on age, race, and sex [24]. Details are outlined in the respective statistical analysis plan.

No interim analyses are planned.

### Trial ethics and governance

This clinical study is being conducted in accordance with the Declaration of Helsinki and in compliance with the study protocol, Good Clinical Practice (GCP), designated SOPs, and local laws and regulations relevant to the country of conduct. The study protocol (version 4.5 for Germany, dated 5 November 2019) was approved by the respective competent authorities and ethic committees involved and used as the basis for this manuscript (country-specific protocol modifications not considered). The study is registered at the European clinical trial database EudraCT database, No.: 2016-002162-30, and in the ClinicalTrials.gov Protocol Registration and Results System, ClinicalTrials.gov ID: NCT03278548.

Written informed consent, in accordance with the origins of the Declaration of Helsinki and the applicable laws of the country, has to be obtained from all patients before entering the study. Informed consent forms for individual countries are available from the corresponding author upon request. The investigator explains the nature, purpose, and risks of the study and provides the patient with a copy of the patient information. The patient will be given sufficient time to consider the study’s implications before deciding whether to participate. There is no compensation for trial participation. All study participants are insured in accordance with the respective national legislations.

Information for the patients on the handling of their personal medical data and on the storage and handling of blood samples is part of the informed consent form. Blood samples that are collected specifically for this clinical study are kept until the end of the clinical study. Any remaining blood samples are destroyed after the completion of the clinical study. Storage of biological specimens for future research is not planned.

In case of any amendments to the protocol that would directly affect the patient’s participation in the study, the informed consent form will be amended, and the patient’s informed re-consent will be obtained, unless the patient has completed all procedures prior to the effectiveness of the corresponding amendments to the protocol.

Protocol amendments will be submitted to the concerned independent ethics committees (IECs) and competent authorities in line with pertinent regulatory requirements.

An independent audit at the study site may take place at any time during or after the study.

Any party (e.g., domestic and foreign regulatory authorities, the sponsor, and/ or authorized representatives of the sponsor such as monitors and auditors) with direct access takes all reasonable precautions within the constraints of the applicable regulatory requirements to maintain the confidentiality of patient identities and sponsor proprietary information. All patient data obtained in the context of the clinical trial are subject to data protection. The patient’s name in addition to other personal data (excluding age and sex) are not to be disclosed by the investigator. The storage of data for statistical assessment shall likewise be performed only under the patient’s study identification. Only the investigator will have the means to identify a patient’s name/other personal details via the study identification. If personal data and study-related documentation are stored and processed, the requirements of pertinent data protection legislation are to be observed.

Data generated in this study is recorded using a computerized system in accordance with applicable regulations. The system generates an individual eCRF for each patient participating in the study. Data entry is done by the respective site investigators, in part supported by study nurses. The responsibilities of the investigator, monitor, and sponsor of this clinical trial as regards to handling of data, storage of data, planning, assessment, and quality assurance are according to the recommendations on “International Conference on Harmonisation Topic E 6 Guideline for Good Clinical Practice.”

Authorized, qualified representatives of the sponsor will visit investigational sites in regular intervals as defined in the monitoring plan, to verify adherence to protocol and local legal requirements, to perform source data verification, and to assist the investigator in study-related activities. As a quality measure for monitoring, respective visits are reported to the study management (e.g., to define suitable corrective and preventive actions). In case the monitor identifies non-adherence to the protocol or legal requirements including data protection and data security requirements, a re-training will be performed, and its adherence will be subsequently strictly controlled.

Withdrawal of individual patients from treatment or from the study respectively could be caused by protocol deviation (e.g., dosing regimen, failure to comply with protocol).

A Data Safety Monitoring Board (DSMB), not involved in study conduct, consisting of two clinicians (one of them appointed as chair) and a biometrician, will monitor the progress of this study with a focus on safety as well as efficacy data.

The criteria for the study termination include unexpected safety concerns assessed by the DSMB.

## Discussion

This study aims to provide data regarding the safety and efficacy of 6% HES 130/0.4 solutions in patients with hypovolemia due to surgery-induced acute blood loss. In contrast to previously published pragmatic studies comparing HES-containing solutions to crystalloid solutions in critically ill patients [25–27], the study design of this clinical trial aims to ensure that the HES 130 containing IP is administered in line with approved dosing recommendations and respecting contraindications. Hemodynamic stabilization is guided in accordance with established hemodynamic algorithms [13–15] and in accordance with the practice of the local investigator.

The primary endpoint of this study is defined as “treatment group mean difference in change from the pre-operative baseline cystatin-C-based eGFR to the eGFR that is calculated from the highest cystatin-C levels measured during post-operative days 1–3,” in line with the scientific advice obtained by the CHMP. eGFR has been shown to be a prognostic indicator of post-operative renal complications, associated with survival [28, 29]. In addition, a decline of eGFR was found to be indicative for acute kidney injury [28, 29]. eGFR can be calculated using defined formulas based on serum creatinine values or serum cystatin-C levels [21]. As cystatin-C was considered a more reliable marker for kidney function than creatinine, the calculation of eGFR in this study is based on serum cystatin-C levels. The primary endpoint of this study is thus a reliable indicator for post-operative renal complications and will allow to compare renal safety of HES 130 administration vs. crystalloid solutions in surgical patients after treatment of acute blood loss.

To demonstrate safety and efficacy for 6% HES 130/0.4 solutions, additional meaningful endpoints (e.g., coagulation, inflammation, hemodynamic variables, serum electrolytes, serum lactate levels, central venous oxygen saturation, length of hospital stay) will be compared between the groups. As major complications including renal impairment are frequently seen in high-risk surgery patients [10, 30], and long-term survival is strongly affected in patients with short-term surgical complications [31–33], a combined secondary endpoint of post-operative complications (including renal) and death will also be analyzed exploratively.

The patient population of this study comprises patients with an increased risk: the age of eligible patients is in the range of > 40– ≤ 85 years because it is known that several risk factors for post-operative morbidity and mortality increase with age. In particular, older age is associated with the development of acute kidney injury (AKI) [18, 34, 35]. Furthermore, we include only patients with ASA Physical Status II–III because it has been shown that concomitant diseases are independent predictors for post-operative morbidity and mortality [18, 19, 30, 36]. Post-operative outcome also depends on the type of surgery: the mortality rate after abdominal surgery is up to 8.4% compared to a general postsurgical mortality rate of up to 1.7% [30, 37, 38]. Moreover, the incidence of post-operative renal dysfunction after abdominal surgery was reported to be approximately 13% [39]. Other types of surgery that are also associated with increased post-operative acute kidney injury are cardiovascular and orthopedic surgeries. In cardiac surgery, however, kidney function is influenced extensively by the use of cardiopulmonary bypass[2, 40], and the mortality rate of older orthopedic surgical patients is usually lower compared to abdominal surgery [19].

In conclusion, this study will allow reliable assessment of the safety of HES 130 administration in surgical patients requiring volume therapy due to surgery-induced acute blood loss. Study results will substantially improve the availability of safety data on HES 130 solutions and allow to evaluate the safety of HES 130 administration in the surgical setting.

### Trial status

This clinical study is currently in the recruitment phase. To update the patient information on regulatory procedures regarding HES products (referral procedure according to Art. 107i of Directive 2001/83/EC, in October 2018 to June 2019), a temporary halt was induced which lasted from January 2019 to July 2019. Recruitment began on September 28, 2017, and the end of recruitment is expected in April 2022.

## Data Availability

It is intended to publish the study results. They may be published as scientific literature in peer-reviewed journals and may also be used in submissions to regulatory authorities. Where necessary, the analyzed study datasets will be shared with regulatory authorities upon request.

## Abbreviations

AKD: Acute kidney disease
AKI: Acute kidney injury
aPTT: Activated partial thromboplastin time
ASA: American Society of Anesthesiologists
Ca^2+^: Calcium
Cl^-^: Chloride
CVP: Central venous pressure
DAP: Diastolic arterial pressure
DSMB: Data Safety Monitoring Board
eCRF: Electronic case report form
eGFR: Estimated glomerular filtration rate
EMA: European Medicines Agency
FAS: Full analysis set
GCP: Good Clinical Practice
Hb: Hemoglobin
HCO_3_^−^: Hydrogen carbonate
Hct: Hematocrit
HES: Hydroxyethyl starch
HR: Heart rate
ICU: Intensive care unit
IEC: Independent ethics committee
INR: International norm ratio
IP: Investigational product
IRTS: Interactive Response Technology System
K^+^: Potassium
LOS: Length of stay
MAP: Mean arterial pressure
Na^+^: Sodium
PADS: Post-Anesthetic Discharge Scoring System
pCO_2_: Carbon dioxide partial pressure
pO_2_: Oxygen partial pressure
POD: Post-operative day
PPS: Per-protocol set
PPV: Pulse pressure variation
RIFLE: Risk, injury, failure, loss, end-stage renal disease
RRT: Renal replacement therapy
SaO_2_: Oxygen saturation
SAEs: Serious adverse events
SAP: Systolic arterial pressure
SCr: Serum creatinine
SOP: Standard operating procedure
SV: Stroke volume
SVI: Stroke volume index
SVV: Stroke volume variation
